# GoFish: A versatile nested PCR strategy for environmental DNA assays for marine vertebrates

**DOI:** 10.1371/journal.pone.0198717

**Published:** 2018-12-11

**Authors:** Mark Y. Stoeckle, Mithun Das Mishu, Zachary Charlop-Powers

**Affiliations:** 1 Program for the Human Environment, The Rockefeller University, New York, New York, United States of America; 2 Hunter College, New York, NY, United States of America; 3 Laboratory of Genetically Encoded Small Molecules, The Rockefeller University, New York, NY, United States of America; University of Guelph, CANADA

## Abstract

Here we describe GoFish, a strategy for single-species environmental DNA (eDNA) presence/absence assays using nested PCR. The assays amplify a mitochondrial 12S rDNA segment with vertebrate metabarcoding primers, followed by nested PCR with M13-tailed, species-specific primers. Sanger sequencing confirms positives detected by gel electrophoresis. We first obtained 12S sequences from 77 fish specimens for 36 northwestern Atlantic taxa not well documented in GenBank. Using these and existing 12S records, we designed GoFish assays for 11 bony fish species common in the lower Hudson River estuary and tested seasonal abundance and habitat preference at two sites. Additional assays detected nine cartilaginous fish species and a marine mammal, bottlenose dolphin, in southern New York Bight. GoFish sensitivity was equivalent to Illumina MiSeq metabarcoding. Unlike quantitative PCR (qPCR), GoFish does not require tissues of target and related species for assay development and a basic thermal cycler is sufficient. Unlike Illumina metabarcoding, indexing and batching samples are unnecessary and advanced bioinformatics expertise is not needed. From water collection to Sanger sequencing results, the assay can be carried out in three days. The main limitations to this approach, which employs metabarcoding primers, are the same as for metabarcoding, namely, inability to distinguish species with shared target sequences and inconsistent amplification of rarer eDNA. In addition, the performance of the 20 assays reported here as compared to other single-species eDNA assays is not known. This approach will be a useful addition to current eDNA methods when analyzing presence/absence of known species, when turnaround time is important, and in educational settings.

## Introduction

DNA profiling of ecological communities was first applied to terrestrial microbes [1,2]. DNA extracted from soil samples—amplified with ribosomal RNA gene primers, cloned, and analyzed by Sanger sequencing—revealed an enormous diversity of uncultured organisms [3]. Whole genome shotgun sequencing provided an alternative culture-independent approach [4]. Combining targeted amplification with high-throughput sequencing eliminated cloning and Sanger sequencing, greatly facilitating microbiome study [5–7]. Around the same time, ancient DNA techniques began to be applied to environmental samples, with recovery of 10,000 years-old to 400,000 years-old plant and animal DNA from fecal samples and sediments [8,9]. The earliest reports examining contemporary materials include differentiating human and domestic sources in sewage-contaminated water [10] and recovery of Arctic fox DNA from snow footprints [11]. Taberlet and colleagues were the first to apply an environmental DNA approach to present-day animal ecology, demonstrating pond water eDNA accurately surveys an invasive frog species [12]. Subsequent work revealed aquatic eDNA detects diverse vertebrates and invertebrates in multiple habitats [13–17]. Aquatic eDNA assays now routinely monitor rare and invasive freshwater species [18–21].

Beginning in 2003, the DNA barcoding initiative firmly demonstrated that most animal species are distinguished by a short stretch of mitochondrial (mt) cytochrome *c* oxidase subunit 1 (COI) gene [22–24]. This led researchers to assess animal communities by “metabarcoding”, i.e., high-throughput sequencing of mtDNA segments amplified from environmental samples [25–28]. The sequence differences that make COI a good identifier of most animal species hobble broad-range primer design [29]. Primers targeting highly conserved regions in vertebrate mitochondrial 12S or 16S ribosomal genes [30–32] often successfully profile aquatic vertebrate communities yet frequently cannot resolve species-level distinctions [33–40]. Multi-gene metabarcoding promises kingdom-wide surveys of eukaryotic diversity [41–45].

Although the growth in technology and approaches have expanded the potential of environmental DNA studies, assessing aquatic vertebrate biodiversity presents several challenges in design and execution. At the lower end of analysis, the development of a single-species qPCR test typically necessitates obtaining tissue samples of the target organism and potential confounding species [46,47], and assays require a specialized thermal cycler. At the higher-throughput end, metabarcoding involves indexing and batching a large number of samples for each sequencing run, and advanced bioinformatics expertise to decode output files. Both approaches have drawbacks that are barriers to adoption. To facilitate wider use, we aimed for an eDNA assay that did not require tissue samples for validation and could be completed in less than a week.

One potential straightforward technique is species-specific amplification followed by gel electrophoresis and Sanger sequencing, as in early eDNA reports [12]. However, our preliminary experiments generated visible products only in samples with a high number of MiSeq reads, indicating low sensitivity. In addition, multiple bands were frequent, likely to interfere with Sanger sequencing. Rather than attempt to optimize conditions for each species-specific PCR, we decided to assess the use of nested PCR, a highly sensitive and specific approach to identifying genetic variants (e.g., [48]). For example, nested PCR improved detection of earthworm eDNA from soil samples archived for more than 30 years [49] and enabled highly sensitive eDNA assays for salmonid fishes and a fresh water mussel [50,51].

We reasoned that nested PCR applied to a metabarcoding target could be a more general approach. Amplification with broad-range metabarcoding primers could provide a “universal” first round, followed by nested PCR with species-specific primers, taking advantage of sequence differences within the amplified segment. Here we test whether this approach detects aquatic vertebrate eDNA in time-series water samples from lower Hudson River estuary and southern New York Bight and compare results to those obtained with MiSeq metabarcoding. Because this assay involves querying amplified material one species at a time, we name it after the children’s card game Go Fish in which a player might ask “do you have any Jacks?”

## Results

### New 12S reference sequences

**Fig 1:**
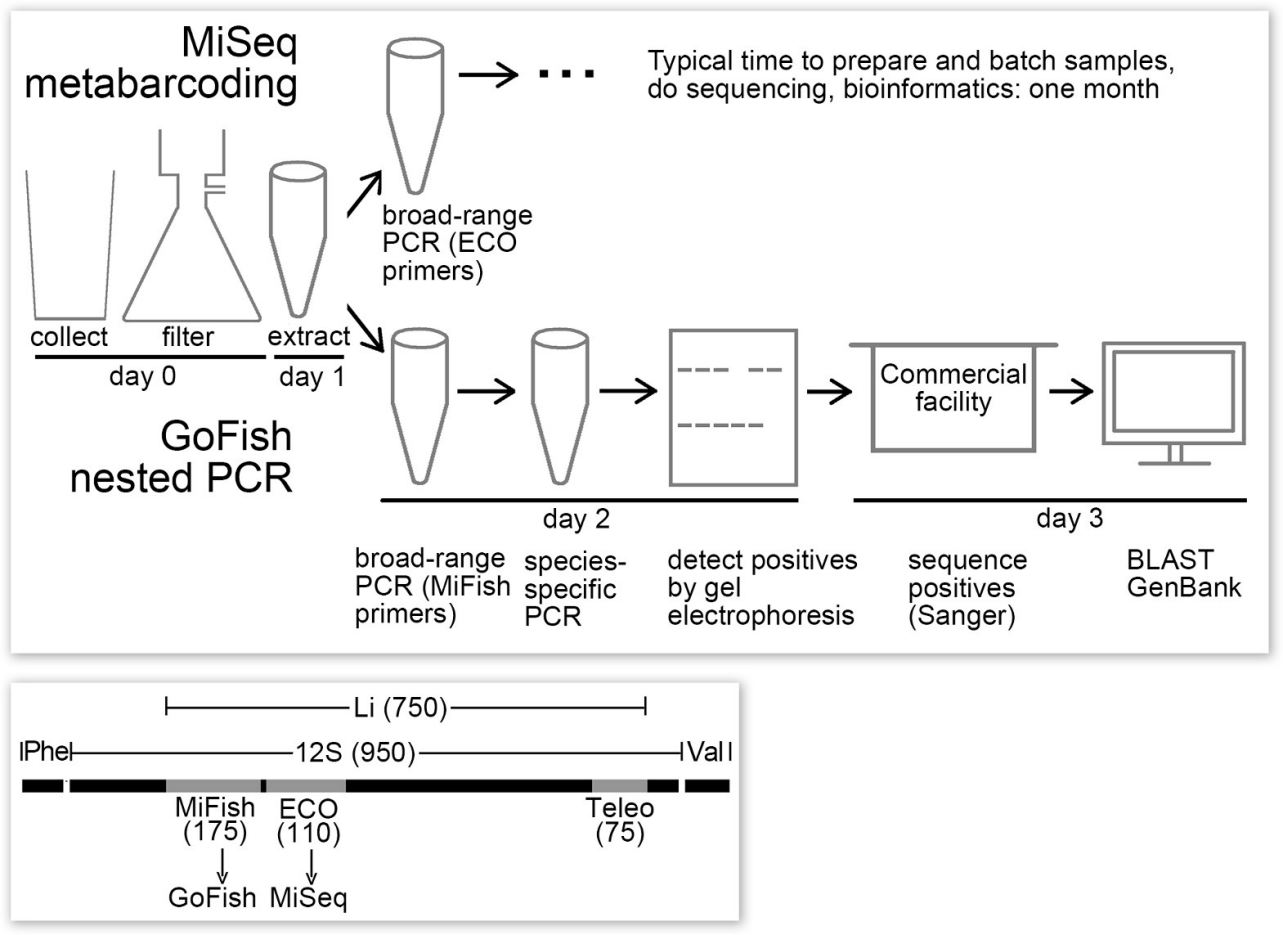
GoFish eDNA assay. Top, schematic of GoFish and MiSeq metabarcoding protocols. Bottom, diagram of 12S and flanking tRNA genes, with locations and sizes of vertebrate metabarcoding targets (MiFish, ECO, Teleo) and the Li segment sequenced from reference specimens as indicated. Typical times for assays are shown; a suitably equipped and staffed laboratory could perform MiSeq metabarcoding in a similar time frame as GoFish.

Regional checklist species [52,53] with absent or incomplete GenBank 12S records were flagged. For bony fishes, we focused on common species; for the much smaller number of cartilaginous fishes we sought specimens from any species with absent or incomplete GenBank 12S records. Seventy-seven specimens representing 36 target species were obtained from NOAA Northeast Spring Trawl Survey, Monmouth University, fish markets, or beach wrack (S1 and S2 Tables). Specimen DNAs were sequenced for a 750-base pair (bp) 12S “Li segment” [54] encompassing three commonly used vertebrate eDNA target sites [31,32,34] (Fig 1), and for 648 bp COI barcode region. COI sequences confirmed taxonomic identifications, showing 99.4% to 100% identity to GenBank reference accessions, excepting that of Northern stargazer (*Astroscopus guttatus*), which at the time of this study had no GenBank COI records (S1 and S2 Tables).

### GoFish nested PCR assay

**Fig 2:**
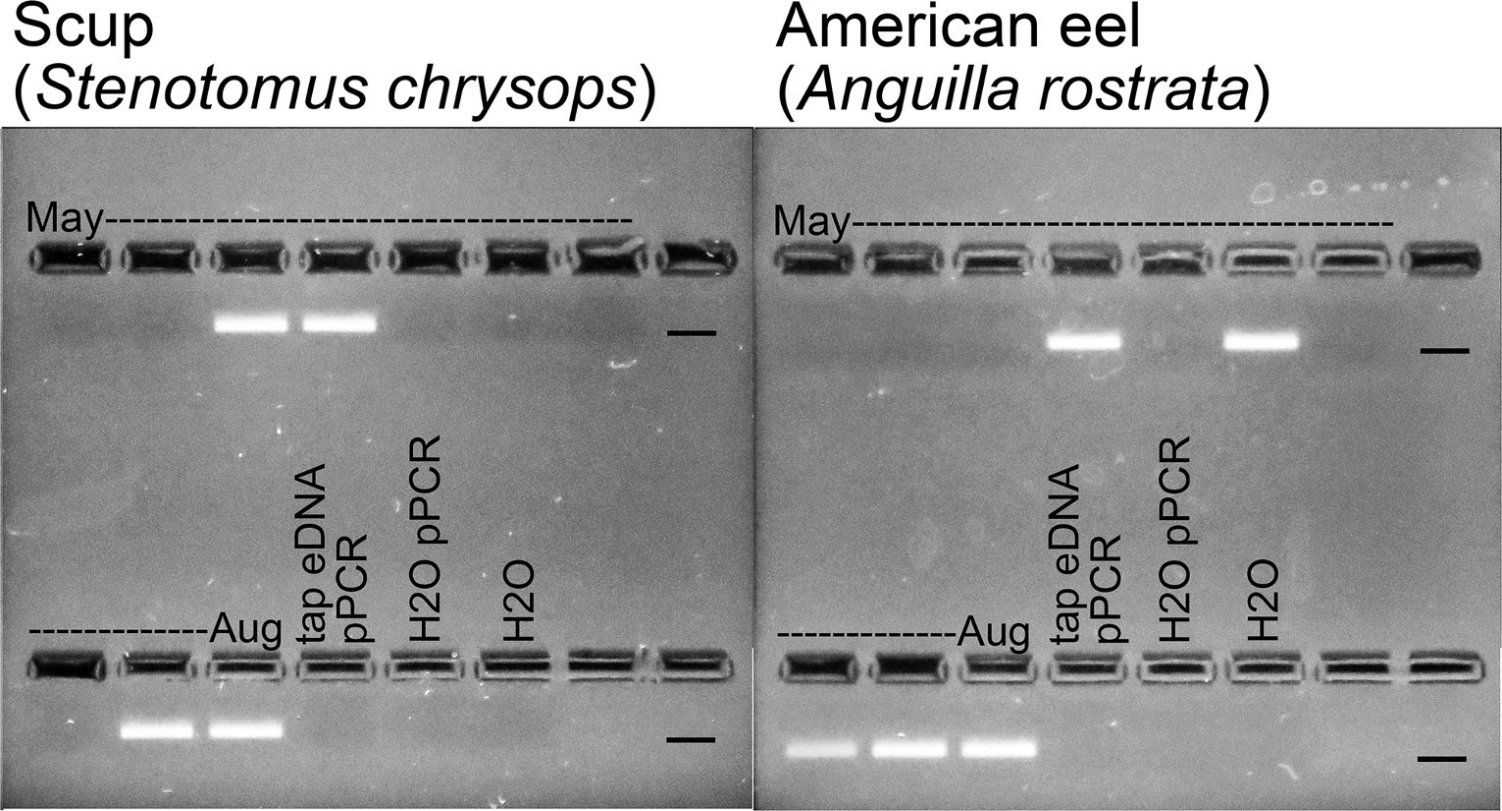
Representative GoFish amplifications visualized on 2.5% agarose gel with SYBER Safe. Lanes bracketed by dates are time series samples from East River site; the last three lanes in each panel are negative controls detailed in Materials and Methods. Marker indicates dye front at approximately 150 bp. Gel positives were sent for Sanger sequencing to confirm target species amplification.

Of three commonly used vertebrate 12S metabarcoding targets (Fig 1), the MiFish segment is longer than the other two and has hypervariable regions near the ends, features facilitating species-specific nested PCR. In addition, by targeting a different segment than what our laboratory uses for MiSeq metabarcoding (12S ECO V5), we aimed to minimize potential cross-contamination between GoFish and metabarcoding assays. First-round PCR for bony fish was done with MiFish primer set [32] (Fig 1, Table 1). The resultant reaction mix, diluted 1:20 in Elution Buffer (10mM Tris pH 8.3, Qiagen) served as input DNA for species-specific PCRs. GoFish primers were designed for 11 fish species that together account for most (92%) lower Hudson River estuary fish eDNA reads (Table 2) [55]. The nested primers generated strong, single bands on gel electrophoresis (Fig 2). In all samples analyzed so far, Sanger sequencing confirmed that GoFish primers correctly amplified the targeted species.

**Table 1:** Broad-range vertebrate 12S primers. PCR parameters and expected amplicon sizes are shown. M13 and Illumina tails in Li primers and ECO V5 primers, respectively, are highlighted in bold.

	Name	Tm (C) (not incl M13 or Illumina tail)	Sequence	Amplicon length incl primers (bp)	Cycles	Annealing temp (C)	Reference
**12S Li segment (Reference specimen sequencing)**							
Bony fish	M13Li-F	56.6	**TGT AAA ACG ACG GCC AGT** GYC GGT AAA AYT CGT GCC AG	760	35	57	[54]
	M13Li-R	60.6	**CAG GAA ACA GCT ATG AC** YCC AAG YGC ACC TTC CGG TA				[54]
Cartilaginous fish	M13Li-S-F	54.6	**TGT AAA ACG ACG GCC AGT** GTT GGT HAA TCT CGT GCC AG	760	35	57	This report
	M13Li-S-R	52.1	**CAG GAA ACA GCT ATG AC** TCC AAG TRC ACT TTC CAG TA				This report
**12S MiFish segment (GoFish first-round PCR)**		** **		** **	** **	** **	** **
Bony fish	MiFish-U-F	58.7	GTC GGT AAA ACT CGT GCC AGC	220	40	60	[32]
	MiFish-U-R	56.7	CAT AGT GGG GTA TCT AAT CCC AGT TTG				[32]
Cartilaginous fish	MiFish-E-F	56.5	GTT GGT AAA TCT CGT GCC AGC	220	40	55	[32]
	MiFish-E2-R	52.2	CAT AGT AGG GTA TCT AAT CCT AGT TTG				This report
Mammals	MiFish-W-F	55.1	GTT GGT AAA TTT CGT GCC AGC	220	40	60	This report
	MiFish-U-R		same as for bony fish				
**12S ECO V5 segment (MiSeq metabarcoding)**							
Bony fish, mammals	ECO-V5-F	50.1	**TCG TCG GCA GCG TCA GAT GTG TAT AAG AGA CAG** ACT GGG ATT AGA TAC CCC	200	40	52	[31]
	ECO-V5-R	49.6	**GTC TCG TGG GCT CGG AGA TGT GTA TAA GAG ACA G** TAG AAC AGG CTC CTC TAG				[31]

**Table 2:** Species-specific GoFish primers. Amplicon sizes, PCR parameters, and target specificity as shown. M13 tails are highlighted in bold. All primer sets were newly designed for this study.

	Primer name	Tm species-specific segment (C)	Primer sequence	Amplicon size incl primers (bp)	Annealing temp (C)	Cycles	Nontarget amplification
**Bony fish**							
American eel (*Anguilla rostrata*)	M13ameeF	47.7	**TGT AAA ACG ACG GCC AGT** GGG CTC AAA TTG ATA TTA CA	175	60	25	N
	M13ameeR	49.5	**CAG GAA ACA GCT ATG AC** C GTG AGT TCA AAG GTG T				
Atlantic menhaden (*Brevoortia tyrannus*)	M13atmeF	48.2	**TGT AAA ACG ACG GCC AGT** GAG TGG TTA TGG AGA ACT	174	60	25	N
	M13atmeR	48.2	**CAG GAA ACA GCT ATG AC** ATC CCA GTT TGT GTC CCG				
Bay anchovy (*Anchoa mitchilli*)	M13baanF	48.3	**TGT AAA ACG ACG GCC AGT** GTG GTT ATG GAA TTC TTT TCT	128	60	25	N
	M13baanR	50.3	**CAG GAA ACA GCT ATG AC** GAT AAA GTC ACT TTC GTG TGA				
Black sea bass (*Centropristis striata*)	M13blsbF	51.2	**TGT AAA ACG ACG GCC AGT** GGG TGG TTA GGA CAT ACT ATT	150	60	25	N
	M13blsbR	51.2	**CAG GAA ACA GCT ATG AC** CTT TCG TGG GTT CAG AAT AAG				
Bluefish (*Pomatomus saltatrix*)	M13blfiF	54.6	**TGT AAA ACG ACG GCC AGT** AGA GTG GTT AAG GAA AGC CTG	148	60	25	N
	M13blfiR	57.1	**CAG GAA ACA GCT ATG AC** TCG TGG GGT CAG GAA TGG				
Cunner (*Tautogolabrus adspersus*)	M13cunnF	54.6	**TGT AAA ACG ACG GCC AGT** GTA AAG AGT GGT TAG GGC AAA CTA	156	65	25	N
	M13cunnR	57.5	**CAG GAA ACA GCT ATG AC** CTC TCG TGG GGT CAG GTG				
Oyster toadfish (*Opsanus tau*)	M13oytoF	52.6	**TGT AAA ACG ACG GCC AGT** CGC GGT TAC ACG AAT GA	192	60	25	N
	M13oytoR	50.3	**CAG GAA ACA GCT ATG AC** ATA GTT TAC GTG GTG TCA AAG				
Scup (*Stenotomus chrysops*)	M13scupF	48.7	**TGT AAA ACG ACG GCC AGT** GGG TGG TTA AGA ATA AAC TAA G	171	60	25	N
	M13scupR	50.1	**CAG GAA ACA GCT ATG AC** AAT CCC AGT TTG TGT CTC				
Seaboard goby (*Gobiosoma ginsburgii*)	M13segoF	52.8	**TGT AAA ACG ACG GCC AGT** GCC CAA GTT GAC AAC TCA	176	60	25	N
	M13segoR	51.7	**CAG GAA ACA GCT ATG AC** CTT TCG TGG GGT CAT ATG TA				
Striped bass (*Morone saxatilis*)	M13stbaF	53	**TGT AAA ACG ACG GCC AGT** GGT TAA GGG CCC AAC TTT TAT	148	65	25	N
	M13stbaR	57.4	**CAG GAA ACA GCT ATG AC** TTT CGT GGG GTC AGG TTT GAG				
Tautog (*Tautoga onitis*)	M13tautF	50.4	**TGT AAA ACG ACG GCC AGT** GTA AAG AGT GGT TAG GAT AAA CAT	155	60	25	N
	M13tautR	55.7	**CAG GAA ACA GCT ATG AC** CTC TCG TGG GGT CAG GTA				
**Sharks, rays, skates**							
Sand tiger shark (*Carcharias taurus*)	M13stshF	50.8	**TGT AAA ACG ACG GCC AGT** CGA GTA ACT TAT ATT AAT ACT TCC	189	60	35	N
	M13stshR	51.6	**CAG GAA ACA GCT ATG AC** TGA CAT CAA GAT TTC TAG TAG				
Sandbar shark (*Carcharhinus plumbeus*)	M13sbshF	51.3	**TGT AAA ACG ACG GCC AGT** CGA GTA ACT CAC ATT AAC ACA C	190	60	35	N
	M13sbshR	50.4	**CAG GAA ACA GCT ATG AC** GTG ACA TCA AGG TTC CTT AG				
Smooth dogfish shark (*Mustelus canis*)	M13smdoF	50.9	**TGT AAA ACG ACG GCC AGT** CGA GTG ACT CAT ATT AAC ACA C	186	60	35	N
	M13smdoR	52.2	**CAG GAA ACA GCT ATG AC** GCA TCA AGG CTC CTT GA				
Bullnose ray (*Myliobatis freminvillei*)	M13buraF	51.4	**TGT AAA ACG ACG GCC AGT** AGG GTG ATT AGA ATT AAT CTC ATC T	159	65	35	N
	M13buraR	50.1	**CAG GAA ACA GCT ATG AC** TGT CGT GAG GTC AAA AAC				
Cownose ray (*Rhinoptera bonasus*)	M13coraF	50.2	**TGT AAA ACG ACG GCC AGT** GGT GAT TAG AAA TAA TCT CAC CA	155	60	35	N
	M13coraR	51.3	**CAG GAA ACA GCT ATG AC** CGT GAG GTC AAA AAT TCT GTT TA				
Roughtail stingray (*Dasyatis centroura*)	M13rostF	50.8	**TGT AAA ACG ACG GCC AGT** ACG AGT GAC ACA AAT TAA TAT CC	189	65	35	N
	M13rostR	50.8	**CAG GAA ACA GCT ATG AC** GTG AGG TCA AAA ACT CTG TTA A				
Spiny butterfly ray (*Gymnura altavela*)	M13sbraF	50.9	**TGT AAA ACG ACG GCC AGT** TAA GGG TGA TTA GAA AAA TCT CAT TT	157	65	35	N
	M13sbra-R	50.6	**CAG GAA ACA GCT ATG AC** AGG TCA AAA ATT CTG TTG TGT				
Clearnose skate (*Raja eglanteria*)	M13clskF	49.2	**TGT AAA ACG ACG GCC AGT** CGA GTA ACT CAT ATT AAT ACT TCA C	175	65	35	Y
	M13clskR	52.6	**CAG GAA ACA GCT ATG AC** GTC GTG AAT TCA AAA GCT CTA TTG				
Little skate (*Leucoraja erinacea*)	M13liskF	51.2	**TGT AAA ACG ACG GCC AGT** CGA GTA ACT CAC ATT AAT ACT TCA C	191	65	35	Y
	M13liskR	52.7	**CAG GAA ACA GCT ATG AC** TGT CGT GAG GTC AAA AGC				
**Marine mammals (*Tursiops truncatus*)**							
Bottlenose dolphin	M13bodoF	49.2	**TGT AAA ACG ACG GCC AGT** TGA CCC AAA CTA ATA GAC AC	187	60	25	N
	M13bodoR	49.6	**CAG GAA ACA GCT ATG AC** TCT TAG TTG TCG TGT ATT CAG				

**Fig 3:**
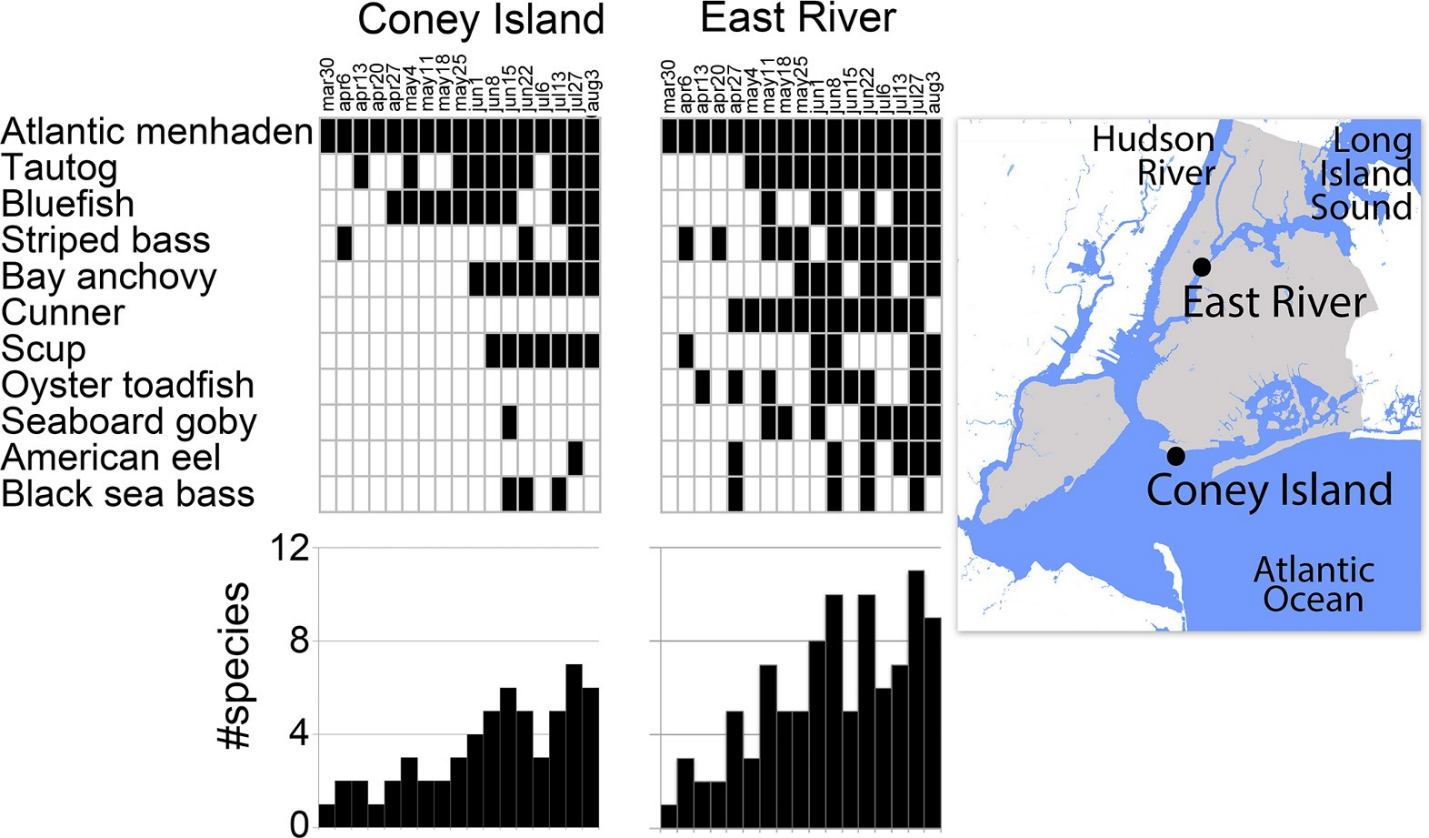
GoFish detections at two lower Hudson estuary locations sampled weekly from March to August 2017. At top, collection dates are shown; black and white rectangles indicate detection and no detection, respectively, with species arranged by decreasing number of positives; at bottom, number of species detected on each date is shown.

We applied these GoFish assays to a four-month time series of water samples collected weekly at two contrasting lower Hudson River estuary locations—a high flow, rocky tidal channel on the east side of Manhattan, and a low-flow, sandy bottom site in outer New York harbor (Fig 3). Species detections increased seasonally at both sites, consistent with historical trawl surveys and a metabarcoding eDNA time series [55]. Despite large tidal flows in the estuary, eDNA differed by site consistent with habitat preferences, with rocky bottom specialists (cunner, oyster toadfish, seaboard goby) more commonly detected in East River than in outer New York harbor.

### Cartilaginous fishes, marine mammals

**Fig 4:**
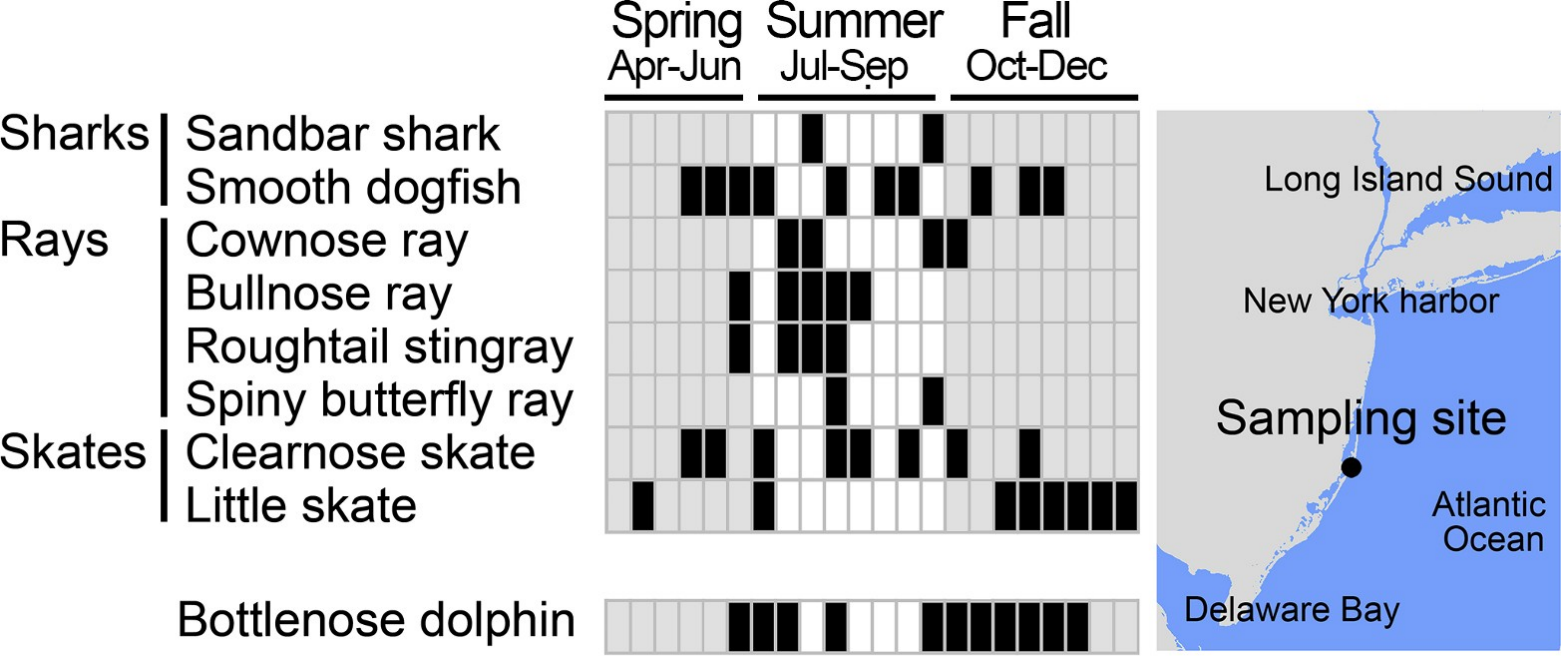
GoFish detections of cartilaginous fishes and bottlenose dolphin eDNA. Water samples were collected at one- to two-week intervals from April to December 2017 in southern New York Bight. Black indicates a GoFish detection, white or gray indicates no detection. The gray shading is added to help visualize demarcation of seasons.

We tested this approach on cartilaginous fishes and marine mammals, groups relatively understudied by eDNA so far [14,36,38,56–58]. First-round MiFish metabarcoding primers were modified to favor cartilaginous fish or mammals (Table 1). Species-specific GoFish amplifications successfully detected three shark species, four rays, and two skates (Table 2). In a nine-month time series of water samples from southern New Jersey, most (64%) cartilaginous fish positives were in summer months (p = 0.017 spring vs. summer; p = 0.001 summer vs. fall, Fisher’s exact test), consistent with seasonal migration patterns (Fig 4) [59]. This statistical analysis excluded little skate (*Leucoraja erinacea*), a cold water species [60] which was detected in spring and fall samples but not in summer. Sanger sequencing confirmed species ID for all gel-positive amplifications, except that clearnose skate (*Raja englanteria*) and little skate primers amplified non-target sequences in some samples. A GoFish assay for bottlenose dolphin (*Tursiops truncatus*) was positive in most summer and fall samples; sequencing verified all (Table 2, Fig 4).

### Comparison to Illumina metabarcoding

**Fig 5:**
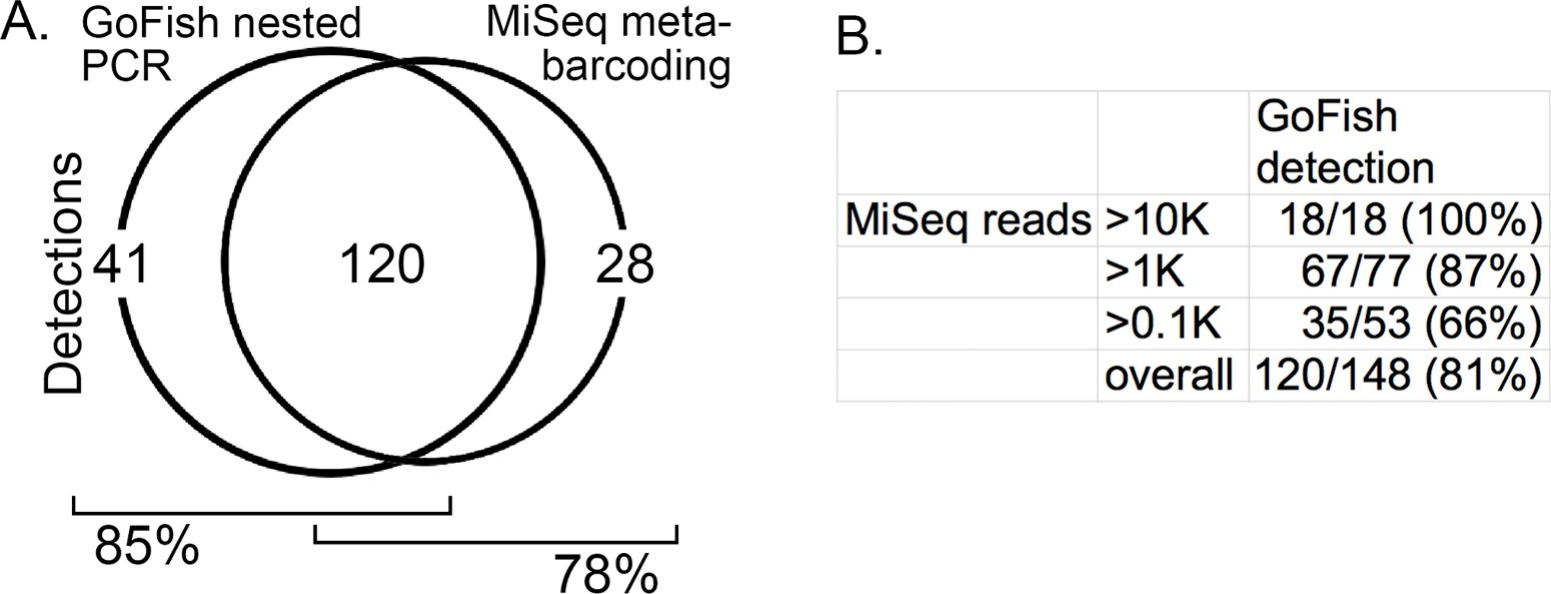
Comparison of GoFish and metabarcoding. A. Number of detections by method for the 11 species and 34 samples shown in Fig 3. A detection refers to a positive GoFish or metabarcoding result for a single species in a single sample, which corresponds to one cell in Fig 3 grid. Of the 161 GoFish detections shown in Fig 3, 120 were also positive by metabarcoding for the same species in the same sample. In addition, there were 28 metabarcoding detections for one of the 11 target species in samples that were negative by GoFish. B. GoFish detections for the 148 metabarcoding positives, sorted by metabarcoding reads per detection.

The New York City time series samples were analyzed by an Illumina MiSeq metabarcoding protocol targeting 12S ECO V5 segment (Fig 1). The apparent sensitivity (method detections/total detections) for both protocols was about 80% (Fig 5). As expected, the proportion of detections negative by GoFish but positive by metabarcoding differed by metabarcoding read number—more abundant eDNAs were detected more consistently than were rarer eDNAs (Fisher’s exact test p = 0.0175 for 0.1K vs. 1K; p = 0.001 for 0.1K vs. 10K).

## Discussion

Here we report species-specific nested PCR eDNA assays for 20 marine fishes and one marine mammal. The GoFish assay can potentially be adapted to detect any vertebrate with a 12S reference sequence; tissue specimens are not necessary. It can be completed in less than a week with standard molecular biology equipment and interpreted with Sanger sequencing-level bioinformatics. A single broad-range amplification suffices for multiple species-specific assays.

To facilitate primer design we sequenced a 12S fragment, covering three commonly analyzed vertebrate eDNA metabarcoding targets, from 77 specimens representing 36 local species, boosting GenBank 12S coverage to 95% of lower Hudson River estuary checklist species [55]. We chose 12S rather than COI barcoding gene because there are excellent broad-range vertebrate primers for several targets in this gene (Fig 1), which is why 12S is standard for vertebrate metabarcoding. A relative disadvantage to this choice is that coverage of vertebrates in GenBank is less for than for COI. However, available metabarcoding COI primers have significant biases against multiple taxa [29], making the gene less suitable for a GoFish strategy, namely, broad-range followed by species-specific amplification.

GoFish is likely not advantageous in cases where a verified single-species qPCR, digital droplet PCR, or real-time PCR assay and appropriate equipment are available. More generally, the performance of the 20 assays reported here as compared to other single-species eDNA assays is not known. A potential concern is that nested PCR, which involves pipetting PCR products to use as templates in new PCRs, is susceptible to contamination. In this regard we note that pipetting PCR products to use as templates in new PCRs is a routine step in metabarcoding—it is how PCR products are typically indexed prior to Illumina sequencing. To date we have performed more than 400 GoFish assays without evidence of contamination, including in work with closely-supervised students. Another potential issue is assay specificity, given that we did not use tissue samples in assay design or verification. Single-species qPCR assays are typically developed by first selecting candidate species-specific primers *in silico* and then testing these against tissue samples from target and related organisms (e.g., [46,47]). GoFish instead uses bioinformatics—Sanger sequencing of positive amplifications—to document specificity. This is equivalent to metabarcoding—a Sanger sequence of a nested PCR product is as specific as an Illumina MiSeq-generated FASTQ sequence. This bioinformatic approach enable us to design and test 20 species-specific assays in a relatively short time—approximately six months—without the tissue samples and resources that other single-species PCR assays typically require. To our knowledge, 20 single-species assays for marine vertebrates is more than have been reported in the combined literature so far.

The main limitations so far are species lacking sequence differences in the target segment (assays require species-specific substitutions in both primer sites and in amplified segment) and inconsistent amplification of rarer eDNAs, which are shortcomings shared with metabarcoding [61]. Several pairs or sets of local species have insufficient MiFish segment sequence differences for GoFish assays. Of note, these include *Alosa* herrings, of commercial and conservation interest: alewife (*A*. *pseudoharengus*), American shad (*A*. *sapidissima*), blueback herring (*A*. *aestivalis*), and hickory shad (*A*. *mediocris*). With the single amplification protocol employed in both assays, GoFish sensitivity was equivalent to that of MiSeq metabarcoding, with dropouts in both assays (Fig 5). As expected, GoFish dropouts were mostly those with lower MiSeq read numbers, and presumably represent rarer eDNAs. Inconsistent amplification of low abundance DNAs was recognized as a hazard early on [12]. If desired, replicate amplification or other PCR enhancement strategies [62] could be applied to GoFish. False-negatives may be inherent to broad-range primers, which “rarely detect lineages accounting for less than 0.05% of the total read count, even after 15 PCR replicates” [63] (also [42]). Looked at more broadly, all ecological survey methods generate false-negatives; site occupancy modeling can help infer true presence/absence [64–67].

We assumed that sequences matching regional species indicated the presence of that species. This could lead to overlooking extralimital occurrences of taxa that possess shared sequences. For instance, the locally abundant Atlantic menhaden (*Brevoortia tyrannus*) shares GoFish target sequences with Gulf menhaden (*B*. *patronus*), found in Gulf of Mexico. More generally, the performance of the assays reported here in other marine regions is not known. Although likely impractical to apply GoFish to the hundreds of fish species typically resident in any given marine region, it may be possible to characterize communities by targeting the smaller number of species that account for the majority of biomass (e.g., Fig 3). The non-labor costs for a GoFish assay were about $15 per sample for one species, and $8 per sample per additional species. One difficult-to-quantify advantage is constrained cross-contamination. Because it is cost-effective to test small sets of samples, a GoFish assay puts fewer results at risk than does high-throughput sequencing. This feature could be particularly valuable in educational settings with less expert performers, instead of putting “all your eggs in one basket” in a MiSeq run.

eDNA promises to help better understand and appreciate ocean life. We believe that GoFish will be a useful addition to eDNA tools when species of interest are known and are relatively few in number, when turnaround time is important, and in educational settings.

## Materials and methods

### New 12S, COI reference sequences

DNA was extracted from tissues using the PowerSoil kit (MoBio). 12S primer sequences and PCR parameters applied to reference specimen DNAs are shown in Table 1. Amplifications were confirmed by agarose gel electrophoresis with SYBER Safe dye (Thermo Fisher Scientific), and PCR clean-up and bidirectional sequencing with M13 primers were done at GENEWIZ. Consensus sequences were assembled in MEGA, using 4Peaks to assess trace files [68,69]. For COI, COI-3 primer cocktail [70], 35 cycles and 55°C annealing were used. Substitute forward primers were employed for specimens that failed to generate high-quality sequences with the COI-3 cocktail [hickory shad, American shad (M13alosaCOI-F, 5’-TGT AAA ACG ACG GCC AGT TCA ACT AAT CAT AAA GAT ATT GGT AC-3’); windowpane flounder (M13wiflCOI-F, 5’-TGT AAA ACG ACG GCC AGT CTA CCA ACC ACA AAG ATA TCG G-3’)]. The newly obtained 12S and COI reference sequences (S1 and S2 Files) are deposited in GenBank (Accession nos. MH377759-MH377835 and MH379020-MH379090, respectively).

### Water collection, filtration, DNA extraction

Water sampling was done under permit from New York City Department of Parks and Recreation at two locations: East River (40.760443, -73.956354), a rocky, high-flow tidal channel on the east side of Manhattan, and Steeplechase Pier, Coney Island (40.569576, -73.983297), a sandy bottom, low-flow location in outer New York Harbor (Fig 3). One-liter surface water samples were collected weekly at both sites from March 31, 2017 to August 3, 2017 (34 samples in total). With authorization from New Jersey Department of Environmental Protection, surface water samples were collected on a barrier island beach (39.741641, -74.112961) about 110 kilometers south of New York City and halfway to Cape May, the southern border of New York Bight (Fig 3). 22 one-liter samples were collected at one- to two-week intervals from April 2, 2017 to December 23, 2017.

Samples were filtered within 1 h of collection or stored at 4°C for up to 48 h beforehand. Water was poured through a paper coffee filter to exclude large particulate matter and then into a filtration apparatus consisting of a 1000 ml side arm flask attached to wall suction, a frittered glass filter holder (Millipore), and a 47 mm, 0.45 μM pore size nylon filter (Millipore). Filters were folded to cover the retained material and stored in 15 ml tubes at -20° C prior to DNA extraction. As negative controls, one-liter samples of laboratory tap water were filtered and DNA extracted using the same equipment and procedures as for environmental samples. DNA was extracted with PowerSoil kit with modifications from the manufacturer’s protocol to accommodate the filter [55]. DNA was eluted with 50 μl Buffer 6 and concentration measured using a Qubit (Thermo Fisher Scientific). Typical yield was 1 μμg to 5 μg DNA per liter water filtered. No animals were housed or experimented upon as part of this study. No endangered or protected species were collected.

### GoFish overview

GoFish protocols were designed for persons familiar with basic molecular biology techniques and access to essential molecular biology laboratory equipment. To facilitate use, we utilized commercial kits and open source software, and standardized PCR and sequencing protocols. Procedures were performed on an open bench following routine molecular biology precautions. Particulars include gloves worn for all laboratory procedures and changed after handling water samples and PCR reactions, filtration equipment scrubbed and rinsed thoroughly after each use with tap water, and pipettors and workspace areas wiped with 10% bleach after use. Unfiltered pipette tips were employed; after each procedure used tips were discarded and collection containers rinsed with 10% bleach. Our aquatic eDNA methods are posted online at protocols.io site (https://dx.doi.org/10.17504/protocols.io.p9gdr3w).

### GoFish first-round 12S amplification with metabarcoding primers

Materials and conditions were as follows: GE Illustra beads in 0.2 ml tubes (8 tube strips); 25 μl reaction volume; 5 μl input DNA; 250 nM each primer; and thermal cycler program of 95°C for 5 m, 40 cycles of [95°C for 20 s, (55°C or 60°C) for 20 s, 72°C for 20 s], and 72°C for 1 m. Primers were obtained from Integrated DNA Technologies (IDT). Different MiFish primer sets targeted bony fish, cartilaginous fish, or marine mammals. Primer sequences and annealing temperatures are shown in Table 1. Tap water eDNA and reagent-grade water were included as negative controls on all amplification sets. After PCR, 5 μl of reaction mixture were run on a 2% agarose gel with SYBER Safe to assess amplification. Rather than affinity bead purification, we diluted the reaction mix 20-fold in Elution Buffer and used 5 μl for nested amplifications, effecting a 100-fold dilution of first-round reaction products. With this protocol, a single broad-range amplification sufficed for 80 species-specific assays.

### GoFish species-specific nested PCR

An alignment of 12S MiFish segment sequences from regional fish species including those obtained in this study, marine mammals, and commonly detected non-marine vertebrates (human, pig, chicken, cow, dog, rat), was generated in MEGA using MUSCLE [68,71], sorted according to a neighbor-joining tree, exported to Excel, and used to generate a matrix showing differences from the consensus [72]. Primers were selected by eye according to desired criteria: two or more nucleotide mismatches against other species at or near the 3’ end, a T_m_ not including M13 tail of 50.0°C to 52.0°C according to IDT website, and diagnostic differences within the amplified segment that confirmed target species detection. G-T or T-G primer-template mismatches were considered relatively permissible and thus less useful for conferring specificity [32]. M13 tails enabled a single primer set to sequence all detections and improved 5’ end reads; the latter was particularly helpful given the short amplicons generated by GoFish primers (Table 2).

Limited customization of cycle number and annealing temperature was applied (Table 2), otherwise amplification parameters were same as for first-round PCR. Default annealing temperature was 60°C; if non-target amplification occurred, primers were tested at 65°C. Three negative controls were included in all runs: the two negative controls from the broad-range PCR, and a reagent-grade water blank; these were negative in all assays. A 5 μl aliquot of each PCR reaction was run on an agarose gel with SYBER Safe (Fig 2); positives were sent to GENEWIZ for cleanup and bidirectional sequencing with M13 primers. Sanger-generated sequences were matched to a local file of 12S reference sequences. This file included 12S sequences of local species already in GenBank and new 12S sequences generated from fish specimens reported in this study. All species assignments were based on 100%, full-length matches.

### Metabarcoding

As a comparison, eDNA samples were also analyzed by MiSeq metabarcoding protocol previously described (Fig 1) [55]. Briefly, DNA samples from PowerSoil extraction were further purified with AMPure XP (Beckman Coulter) and suspended in 50 μl of Elution Buffer. 5 μl of each sample were amplified using broad-range primers that target 12S ECO V5 segment in bony fish and mammals. Primer sequences and customized amplification parameters are given in Table 1. Tap water eDNA and reagent-grade water negative controls were included in all sets. 5 μl of each reaction were run on a 2% agarose gel with SYBR Safe dye. Some negative controls gave faint bands; with MiSeq, these turned out to be human or domestic animal DNA, commonly observed in eDNA work [73] (S3 Table). PCR products were diluted 1:20 in Elution Buffer and Nextera index primers (Illumina) were added following the standardized amplification protocol with 12 cycles and annealing temperature 55°C. 5 μl of each reaction were run on a 2% agarose gel with SYBR Safe dye to confirm amplification. Indexed PCR libraries were pooled, treated with AMPure XP, and adjusted to 5.4 ng/μl (30 nM assuming 270 bp amplicon) according to Qubit. Sequencing was done at GENEWIZ on an Illumina MiSeq (2 x 150 bp). 34 experimental and 13 control libraries, plus other samples not reported here, were analyzed in two runs with 92 and 96 libraries per run, respectively. PhiX spike-in was not employed. Original FASTQ files with metadata are deposited in NCBI Sequence Read Archive (NCBI BioProject ID PRJNA358446).

Bioinformatic analysis was performed using DADA2, which identifies all unique sequences rather than lumping according to threshold criteria [74]. DADA2-generated OTU tables and FASTA files of unique sequences are in Supporting Information (S3 Table, S3 and S4 Files). All OTU species assignments were made by 100%, full-length BLAST matches to a local file of reference sequences already in GenBank and reference sequences generated from fish specimens reported in this study. In addition, all OTU sequences, including those without matches to local reference file, were submitted to GenBank using BLAST and alignments checked by eye to confirm assignments. Detections representing less than 0.1% of total reads for that OTU sequence were excluded to minimize mis-assigned reads. After filtering, average total reads/marine sample were 68,241 (range 4,110 to 238,042); average fish reads were 24,598 (range 0 to 138,187) (S3 Table). Tap water eDNA and reagent-grade water controls were negative for fish reads after filtering.

## Supporting information

S1 Table Bony fish specimens analyzed for 12S, COI. (TIF)

S2 Table Cartilaginous fish specimens analyzed for 12S, COI. (TIF)

S3 Table DADA2 OTU tables. (XLSX)

S1 File New 12S reference sequences. (FAS)

S2 File New COI reference sequences. (FAS)

S3 File DADA2 FASTA file for MiSeq run jun2017. (FAS)

S4 File DADA2 FASTA file for MiSeq run oct2017. (FAS)
