# Supplementary figures and images for: GoFish: A versatile nested PCR strategy for environmental DNA assays for marine vertebrates
Source: PLoS One. 2018 Dec 11;13(12):e0198717. doi: 10.1371/journal.pone.0198717 (PMC6289459; doi:10.1371/journal.pone.0198717)

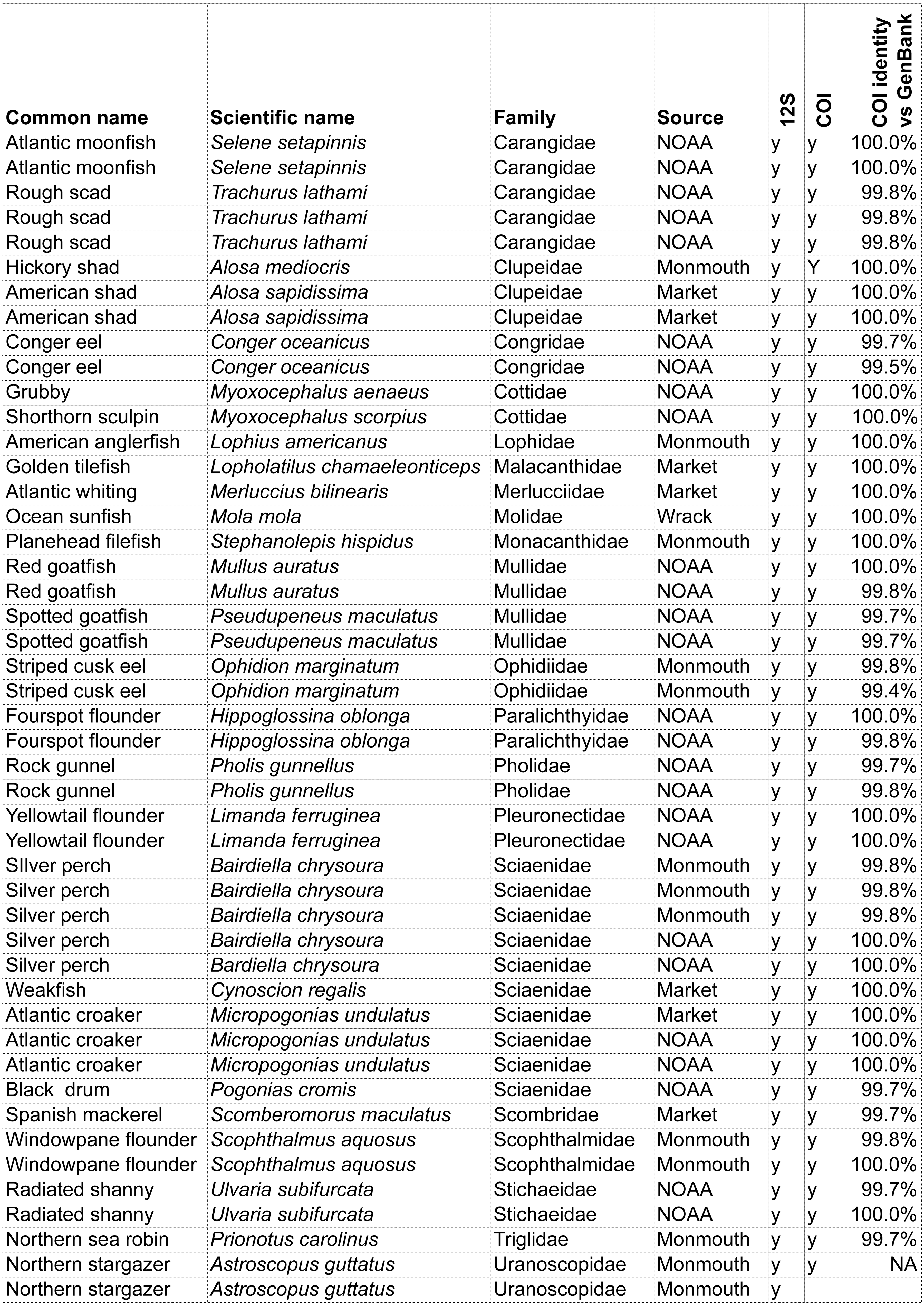

Supplement: S1 Table — (TIF) [file pone.0198717.s001.tif]

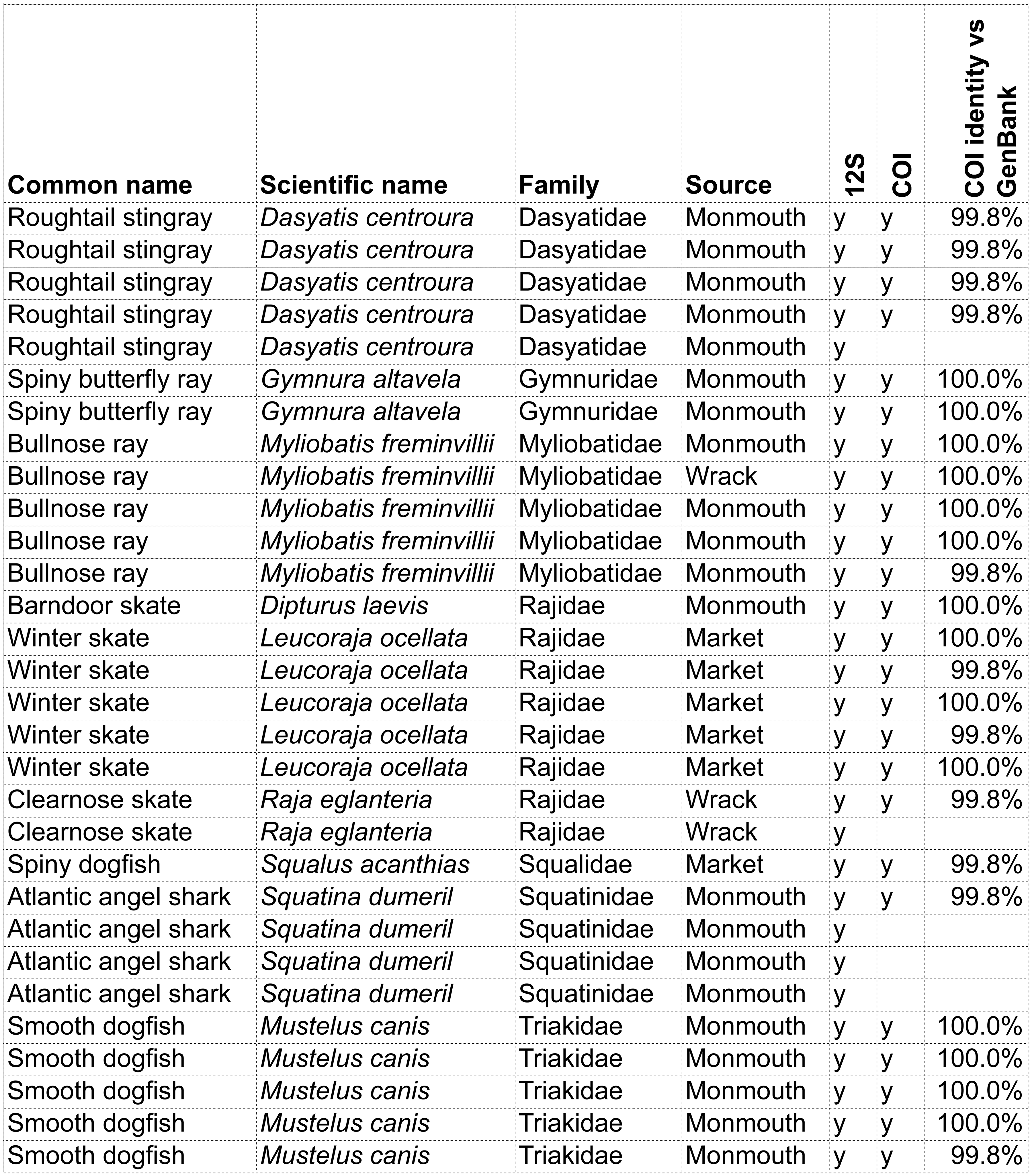

Supplement: S2 Table — (TIF) [file pone.0198717.s002.tif]
